# Anti-human Interleukin(IL)-4 Clone 8D4-8 Cross-Reacts With Myosin-9 Associated With Apoptotic Cells and Should Not Be Used for Flow Cytometry Applications Querying IL-4 Expression

**DOI:** 10.3389/fcell.2019.00046

**Published:** 2019-04-09

**Authors:** Robert Z. Harms, Kiana Borengasser, Vikas Kumar, Nora Sarvetnick

**Affiliations:** ^1^Department of Surgery-Transplant, University of Nebraska Medical Center, Omaha, NE, United States; ^2^Mass Spectrometry and Proteomics Core Facility, University of Nebraska Medical Center, Omaha, NE, United States; ^3^Mary and Dick Holland Regenerative Medicine Program, University of Nebraska Medical Center, Omaha, NE, United States

**Keywords:** interleukin-4, myosin-9, cross-reacting antibodies, 8D4-8, apoptosis, flow cytometry, IL-4

## Abstract

Interleukin(IL)-4 is produced by T cells and other leukocytes and is a critical mediator of monocyte and B cell responses. During routine flow cytometry panel validation for the investigation of intracellular cytokines, we observed unique IL-4 expression patterns associated with the widely available monoclonal antibody 8D4-8. Namely, IL-4 (8D4-8) expression was observed in the absence of cellular activation and enhanced following staurosporine exposure. Mass spectrometry analysis of immunoprecipitates from peripheral blood lymphocytes (PBL) revealed that 8D4-8 cross-reacts with the ubiquitous cytoskeletal protein myosin-9. We confirmed these results by western blotting immunoprecipitates, using immunofluorescence among staurosporine-treated Caco-2 cells, and by surface-labeling PBL for 8D4-8 and myosin-9 and analyzing by flow cytometry. Although previously reported from several independent groups, we found no evidence to support the hypothesis that IL-4 is produced by apoptotic cells. Rather, this appears to have been myosin-9. Our data indicate clone 8D4-8 should not be used in the flow cytometric study of IL-4. Furthermore, our work calls for a reevaluation of previous flow cytometric studies that have used this clone for IL-4 analysis and highlights the importance of validation in antibody-based assays.

## Introduction

The cytokine interleukin 4 (IL-4, b-cell stimulating factor 1) is produced by T cell subsets, mast cells, and basophils and participates in B cell activation, immunoglobulin class switching, and monocyte maturation and polarization (Te Velde et al., 1988; Tadmori et al., 1989; Bradding et al., 1992; Le Gros and Erard, 1994; Mueller et al., 1994; Jung et al., 1995; Blotta et al., 1996; Hart et al., 1999; Tangye et al., 2002). At least 3 isoforms of IL-4 are thought to exist. The canonical sequence generates a functional protein of approximately 17 kD (Yoshizaki et al., 1983; Le et al., 1988; Apweiler et al., 2004). Following the discovery of the full-length protein was the identification of a truncated form missing exon 2 which appears to possess limited bioactivity (Alms et al., 1996; Atamas et al., 1996). More recently, an apoptosis-associated isoform was described (Ledru et al., 2003). This isoform was found to be not secreted and its bioactivity has not been identified.

The flow cytometric analysis of IL-4 expression has allowed for the characterization of Th2-type responses in a variety of settings (Nakayama et al., 2017). Such analyses have been enabled by the generation and commercial availability of several monoclonal antibodies targeting the protein. Of these, clone 8D4-8 was originally described in 1991 from hybridomas derived from Balb/c mice immunized with recombinant human IL-4 (Bird et al., 1991). In this report, the IL-4 used for immunization had been cloned and expressed in yeast and modified to prevent glycosylation. Clone 8D4-8 was found to bind human IL-4 expressed by bacterial, fungal, and mammalian systems. One of the earliest reports using 8D4-8 in flow cytometry was in 1995, in a pivotal methods publication (Prussin and Metcalfe, 1995). Currently, the clone is widely available from multiple commercial sources in a myriad of formats.

An unfortunate reality of any antibody-based assay is the problem of cross-reactivity (deBruin et al., 2010; Benicky et al., 2012; Hansson et al., 2013). This can lead to spurious results and gross misinterpretations of biological phenomena. Here, we describe the outcome of a recent flow cytometric panel validation which revealed unique IL-4 expression associated with clone 8D4-8, which was not observed by 4 other monoclonal antibodies targeting IL-4. Upon closer examination, we found that clone 8D4-8 cross-reacts with myosin-9, a ubiquitous cytoskeletal protein (Pecci et al., 2018). This cross-reaction is associated with cellular apoptosis, generates misleading results, and indicates clone 8D4-8 should be avoided in flow cytometric applications investigating IL-4 expression.

## Materials and Methods

### Cells

We acquired human peripheral blood lymphocytes (PBL) from the UNMC elutriation core facility. PBL were rested overnight in X-VIVO-15 media (Lonza) supplemented with 2% v/v human AB serum (MP Biomedicals), 5 pg/mL IL-2(Cell Sciences), and 20 pg/mL IL-7 (Biolegend) at 37°C with 5% CO_2_ prior to experimentation. We purchased Caco-2 cells (HTB-37) from the ATCC. Caco-2 cells were cultured at 37°C with 5% CO_2_ in minimal essential media (MEM, Hyclone) supplemented with 20% v/v fetal bovine serum (Hyclone), 2 mM L-glutamine (Corning), 1× non-essential amino acids (Hyclone), 1 mM sodium pyruvate (Gibco), 500 units/mL pencillin and 500 μg/mL streptomycin (Gibco). Staurosporine (Thermo Fisher Scientific) was used at 1 μM or stated concentrations.

### Antibodies

We used the following primary antibodies: IL-4 clone 8D4-8 (PE and unlabeled from BioLegend; BrilliantViolet 421, BD Biosciences), IL-4 clone 3010.211 (PE, BD Biosciences), IL-4 clone 4D9 (PE, Beckman Coulter), IL-4 clone REA895 (PE, Miltenyi), IL-4 clone MP4-25D2 (PE, BioLegend), IFN-γ (4S.B3, PE, Biolegend), CD3 (SK7, BrilliantUltraviolet 737, BD Biosciences and UCHT1, FITC, Biolegend), CD4 (RPA-T4, PE-CF594, BD Biosciences), CD45RA (HI100, APC, Biolegend), CD8 (RPA-T8, BrilliantViolet 510, BD Biosciences), CD161 (HP-3G10, BrilliantViolet 421, BioLegend), Vα7.2 (3C10, FITC, BioLegend), myosin-9 (EPR8965, unlabeled, AbCam), myosin-9 (polyclonal EB09020, unlabeled, Everest Biotech), rat IgG1 isotype (eBRG1, PE, eBioscience), mouse IgG1 isotype (MOPC-21, PE, Biolegend), and human IgG1 isotype (QA16A12, PE, Biolegend).

### Flow Cytometry

To induce cytokine production, PBL were stimulated with Cell Stimulation Cocktail (a mixture of phorbol 12-myristate 13-acetate (PMA), ionomycin, brefeldin A, and monensin from eBioscience) or brefeldin A and monensin only [protein transport inhibitors (PTI) from Biolegend] according to manufacturers’ instructions for 5.5 h. Cells were then washed with PBS, counted with a haemocytometer and labeled with Live/Dead UV Blue (Thermo Fisher Scientific) according to manufacturer’s protocol. After washing with flow cytometry staining buffer (FCSB: 0.75% w/v BSA, 1 mM EDTA, 0.05% NaN_3_ in PBS), Fc receptors were blocked with human IgG (Sigma Aldrich). Surface antibodies were added to cells and incubated for 30 min at 4°C in the dark. Cells were washed, fixed with 3% PFA for 30 min at room temperature in the dark. Cell were then washed with Permeabilization buffer (BioLegend), blocked with human, mouse and rat IgG (Jackson Immunoresearch), then labeled with intracellular antibodies for 30 min at room temperature in the dark. Cells were washed with permeabilization buffer, then FCSB before being resuspended in FCSB prior to analysis. Samples were analyzed with a BD LSR II and data were analyzed with FlowJo (v10 TreeStar). For Figure 2B, frequencies were normalized using natural log transformations. They were then compared using one-way analysis of variance (ANOVA). *P* values < 0.05 were considered significant. Statistical tests and associated figures were completed using GraphPad Prism version 6.03.

For myosin-9 and 8D4-8 co-staining, after Live/Dead labeling cells were blocked with 10% donkey serum (Equitech) supplemented with human and mouse IgG. PE-conjugated 8D4-8 and unlabeled myosin-9 (EPR8965) were surface labeled as described above. Cells were washed and incubated with donkey anti-rabbit IgG conjugated to DyLight649 (BioLegend) for 20 min in the dark at 4°C. Cells were then washed, fixed, and analyzed as above.

### Western Blot

We produced protein lysates using radioimmunoprecipitation assay (RIPA) buffer (25 mM Tris–HCl pH 7.6, 150 mM NaCl, 5 mM EDTA, 1% NP-40, 1% sodium deoxycholate, and 0.1% SDS) supplemented with protease inhibitor cocktail III and 0.5 mM PMSF (Research Products International). Cellular disruption was aided by forcing suspensions through a 27 gauge needle repeatedly. We then spun the lysates at 21,000 × *g* for 10 min at 4°C. We determined protein concentrations using the Pierce Rapid Gold BCA Protein Assay kit (Thermo Fisher Scientific) and Pierce Bovine Serum Albumin standards (Thermo Fisher Scientific) to generate the standard curve. Lysates were diluted into Laemlli buffer supplemented with 100 mM DTT (Research Products International) and heated for 15 min at 65°C. 20–35 μg of soluble lysate was loaded per well and recombinant human IL-4 (BioLegend) was loaded at 300 pg per well as a positive control. Precision Plus All Blue standards (BioRad) were used to approximate molecular weight. Proteins were separated using SDS-PAGE with either 14 or 7.5% gels, then transferred onto 0.45 μm PVDF (Thermo Fisher Scientific) in 5% v/v MeOH Towbin buffer overnight at 0.11 A using the Mini Trans-Blot cell (BioRad). Following transfer, membranes were blocked for 2 h with 5% milk in 0.1% Tween 20 v/v tris-buffered saline (TBST). Primary antibodies were diluted in 3% w/v BSA in TBST and incubated with membranes overnight at 4°C with rocking. Purified 8D4-8 was used at 1 μg/mL, GAPDH at 20 ng/mL, myosin-9 (EPR8965) at 96 ng/mL, and myosin-9 (EB09020) at 500 ng/mL. Membranes were then washed thoroughly with TBST and probed with secondary antibodies (Jackson Immunoresearch). Horseradish peroxidase-conjugated Donkey anti-goat, anti-rabbit, and anti-mouse IgG were used at 1:40,000, 1:100,000, and 1:100,000, respectively. Supersignal West Pico Plus and Supersignal West Femto Maximum Sensitivity Chemiluminescent substrates (Thermo Fisher Scientific) were used for detection. Membranes were exposed for 10 s to 1 min onto autoradiography film, then developed using an Optimax Film Processor (Protec). Films were then digitized and annotated using a BioRad Gel Doc system. Blots were reprobed for loading controls following washing in mild stripping buffer (0.1% w/v SDS, 1.5%w/v glycine, 1%v/v Tween 20 pH2.2).

### Immunofluorescence

Following trypsinization at subculturing, Caco-2 cells were seeded onto Nunc LabTek Permanox chamber slides (Thermo Fisher Scientific) and allowed to attach and expand for 2 days. We then removed old media and replaced with fresh media (control) or fresh media supplemented with 1 μM staurosporine and cultured overnight. We then removed media and washed slides gently in PBS. Cells were fixed in 3% w/v PFA for 20 min at room temperature, washed in PBS, and then blocked in 10% donkey serum (Equitech) with 0.1% v/v Triton X-100 (Sigma Aldrich) for 1 h. We diluted primary antibodies in FCSB + 0.1% v/v Triton X-100 and incubated at room temperature for 2 h. Clone 8D4-8 was used at 10 μg/mL and myosin -9 clone EPR8965 was used 500 ng/mL. Control chambers were incubated with antibody diluent only. Following primary incubation, slides were washed thoroughly with PBS and incubated with secondary antibodies for 1 h at room temperature in the dark. Donkey anti-rabbit IgG conjugated to CF568 and donkey anti-mouse IgG conjugated to biotin (Biotium) were used at 3 μg/mL. Slides were then washed with PBS and cultured for 30 min with streptavidin-conjugated CF488A(Biotium) at 8 μg/mL. Following final washing, coverslips were mounted with ProLong Diamond Antifade Mountant with DAPI (Thermo Fisher Scientific). Images were taken using a Zeiss Axio Imager.A2 with an Axiocam 503 mono camera and Zen software. Images were merged and prepared using Fiji (Schindelin et al., 2012).

### Immunoprecipitation

Protein lysates from control and staurosporine-treated PBL were prepared as described above. Lysates (1 mg in 1 mL RIPA) were precleared by adding 1 μg ChromPure mouse IgG (Jackson Immunoresearch) and 60 μL Protein A/G plus Agarose (Santa Cruz Biotechnology) and rotating for 1 h at 4°C. Agarose was pelleted and supernatents were transferred to new tubes. 10 μg 8D4-8 was added and samples were incubated with rotation for 1 h at 4°C after which 60 μL Protein A/G plus Agarose was added and rotation was allowed to continue overnight. Agarose was then pelleted and washed in 10% RIPA in PBS four times. After the final wash, agarose was resuspended in 40 μL Laemlli buffer with 100 mM DTT, heated as above, centrifuged, then 20 μL of immunoprecipitate was separated using SDS-PAGE. After electrophoresis, gels were fixed overnight in 45% methanol, 10% glacial acetic acid. Fixed gels were stained with Coomassie R-250, destained, and ∼100 kD bands were excised and stored in MilliQ water (Millipore) prior to digestion.

### Mass Spectrometry

The stained protein band corresponding to the protein of interest was processed for in-gel digestion at the UNMC proteomics facility using the protocol of Shevchenko et al. (2006). Extracted peptides were re-suspended in 2% acetonitrile (ACN) and 0.1% formic acid (FA) and loaded onto trap column Acclaim PepMap 100 75 μm × 2 cm C18 LC Columns (Thermo Fisher Scientific) at flow rate of 4 μl/min. These were then separated with a Thermo RSLC Ultimate 3000 (Thermo Fisher Scientific) on a Thermo Easy-Spray PepMap RSLC C18 75 μm × 50 cm C-18 2 μm column (Thermo Fisher Scientific) with a step gradient of 4–25% solvent B (0.1% FA in 80% ACN) from 10 to 22 min and 25–35% solvent B for 22–32 min at 300 nL/min and 50°C with a 70 min total run time. Eluted peptides were analyzed by a Thermo Orbitrap Fusion Lumos Tribrid (Thermo Fisher Scientific) mass spectrometer in data dependent acquisition mode. A survey full scan MS (from m/z 350–1800) was acquired in the Orbitrap with a resolution of 120,000. The AGC target for MS1 was set as 4 × 10^5^ and ion filling time set as 100 ms. The most intense ions with charge state 2–6 were isolated in 3 s cycle and fragmented using HCD fragmentation with 40% normalized collision energy and detected at a mass resolution of 30,000 at 200 m/z. The AGC target for MS/MS was set as 5 × 10^4^ and ion filling time set 60 ms dynamic exclusion was set for 30 s with a 10 ppm mass window. Protein identification was performed by searching MS/MS data against the Swiss-Prot human protein database downloaded on Aug 20, 2018. The search was set up for full tryptic peptides with a maximum of two missed cleavage sites. Acetylation of protein N-terminus and oxidized methionine were included as variable modifications and carbamidomethylation of cysteine was set as fixed modification. The precursor mass tolerance threshold was set 10 ppm and maximum fragment mass error was 0.02 Da. Qualitative analysis was performed using PEAKS 8.5 software. The significance threshold of the ion score was calculated based on a false discovery rate of ≤1%.

## Results

**FIGURE 1:**
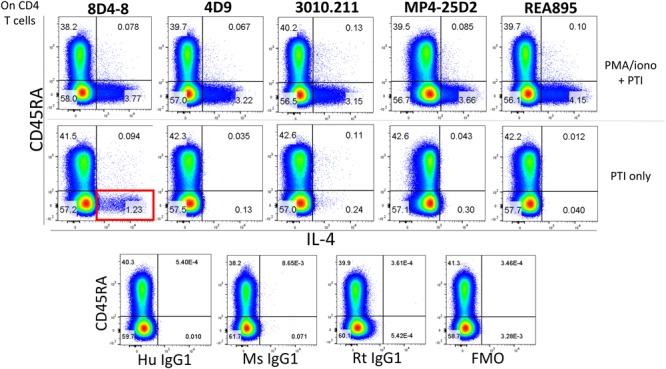
Anti-IL-4 clone 8D4-8 uniquely detects a population of events in the absence of PMA/ionomycin stimulation. CD4 T cells were identified as CD4+, CD8-, CD3+, Live/Dead-, singlet events (not shown). Anti-IL-4 clones 8D4-8, 4D9, 3010.211, MP4-25D2, and REA895 identify comparably sized subsets among CD45RA- CD4 T cells following PMA/ionomycin stimulation. However, clone 8D4-8 identifies a sizeable population of events (red box) following culture with only brefeldin A and monensin (protein transport inhibitors, PTI). Isotype and fluorescence minus one (FMO) controls are shown at bottom for reference. Mouse IgG1 is the isotype for 8D4-8, 4D9, and 3010.211, rat IgG1 is the isotype for MP4-25D2, and human IgG1 is the isotype for REA895. Data shown are representative from 3 experiments from 3 donors.

**FIGURE 2:**
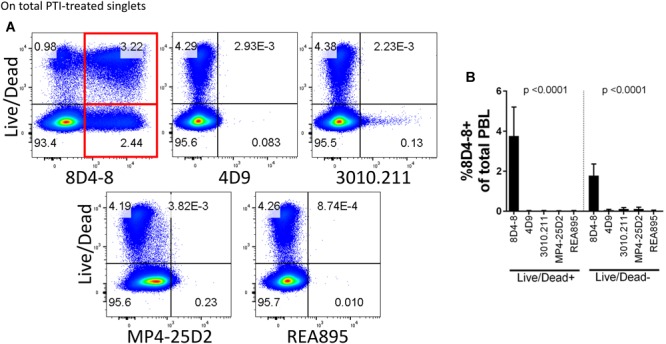
Clone 8D4-8 expression is enriched among Live/Dead+ and Live/Dead- cells. PBL were pregated to remove doublets (not shown), and then examined to compare IL-4 expression with Live/Dead following treatment with protein transport inhibitors (PTI). **(A,B)** Clone 8D4-8 uniquely detects a sizeable population Live/Dead+ cells, as well as a subset of Live/Dead- events. In comparison, clones 4D9, 3010.211, REA895, and MP4-25D2 show negligible binding among Live/Dead+/- events. Data shown are representative from 3 experiments from 3 donors. Bars represent mean values and standard deviation. Significance was determined by ANOVA.

In the process of flow cytometry panel validation, we compared IL-4 expression among total CD4 T cells following PMA/ionomycin stimulation using 5 separate monoclonal antibodies: clones 8D4-8, MP4-25D2, 4D9, REA895, and 3010.211. Clones 4D9, REA895, 3010.211 and MP4-25D2 generated clean populations of IL-4 positive events that were specific to PMA/ionomycin-stimulated cells and enriched in the antigen-experienced CD45RA- subset (Figure 1). Alternatively, clone 8D4-8 appeared somewhat less specific. Clone 8D4-8 uniquely bound a population of IL-4+ events in the absence of PMA/ionomycin stimulation (Figure 1). Further analysis of total PBL revealed that these events were both Live/Dead+/- and significantly higher with 8D4-8 than with the remaining clones (Figure 2). From these results, we concluded that 8D4-8 performed differently than the other clones. This led us to wonder if a unique IL-4 isoform was actually detected by 8D4-8.

**FIGURE 3:**
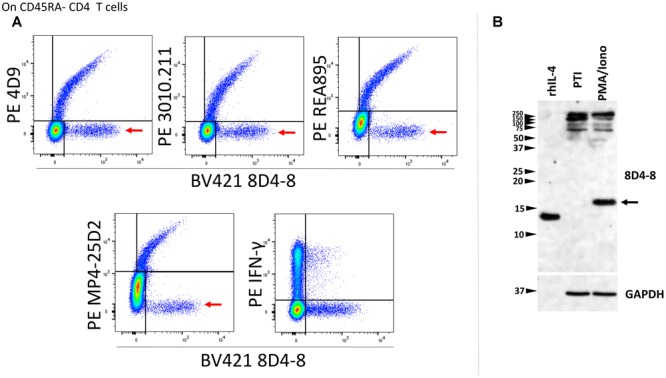
Clone 8D4-8 detects IL-4, yet also identifies a unique target. CD45RA- CD4 T cells were identified as CD45RA-, CD4+, CD8-, CD3+, singlet events (not shown). **(A)** When co-labeled with other IL-4 clones, 8D4-8 detects 2 populations of cells: one which is apparently IL-4 and another unknown target which is limited to 8D4-8. Importantly, clones 4D9, 3010.211, REA895, and MP4-25D2 do not detect unique populations. 8D4-8 was also compared with IFN-γ as a control. Data shown are representative of 3 separate experiment from 3 donors. **(B)** Western blotting of recombinant human IL-4 (rhIL-4), protein transport inhibitor (PTI)-treated, and PMA/ionomycin with PTI- treated cell lysates demonstrate 8D4-8 is capable of detecting recombinant IL-4 as well as natural IL-4 (black arrow). Multiple high molecular weight bands are present in both treatments. Western blot data are representative of 5 stimulations and experiments from 5 donors.

We then revised our flow cytometry panels to include 2 distinct IL-4 clones, or 8D4-8 with IFN-γ as a control. We tested BV421-conjugated 8D4-8 paired with PE-conjugated 4D9, REA895, MP4-25D2, or 3010.211, as well as with PE-conjugated IFN-γ (clone 4S.B3). Interestingly, we observed each pair of anti-IL-4 antibodies bound a double positive subset (Figure 3A). However, clone 8D4-8 was the only antibody that bound a clone-specific population (Figure 3A red arrows). The results from these analyses indicate that 8D4-8 appears to bind similar targets as the other 4 clones, yet is able to detect a unique population of cells.

As alternate IL-4 isoforms have been previously described but less well characterized, we sought to analyze lymphocyte lysates by western blotting in order to determine which IL-4 isoform may be detected by 8D4-8. Here, we compared PMA/ionomycin-treated PBL with PBL given only PTIs. Probing with 8D4-8, we observed a strong band around 17 kD associated with PMA/ionomycin stimulation (Figure 3B). However, there were no demonstrable bands less than 20 kD associated with PTI treatment. Interestingly, both treatments generated bands between 250 and 100 kD. Thus, we found no evidence for known IL-4 isoforms, which should be lower molecular weight than the canonical form.

**FIGURE 4:**
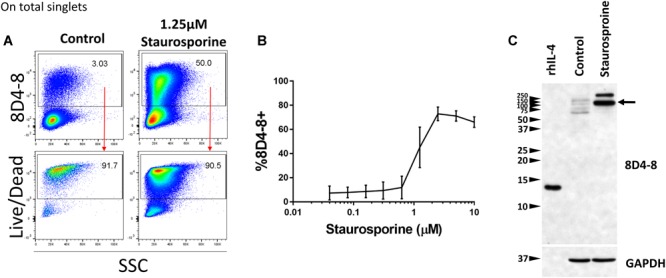
Staurosporine treatment increases the expression of the 8D4-8 target. PBL were pregated to remove doublets (not shown). **(A)** Representative scatter plots showing control and 1.25 μM staurosporine-treated PBL. Staurosporine-treated PBL show increased expression of the 8D4-8 target, and, just like in control, these cells are mostly Live/Dead+. **(B)** Increasing concentrations of staurosporine induce the expression of the 8D4-8 target. In the graph, points are averages and error bars represent standard deviation. For **(A,B)** data are representative of 3 experiments from 3 donors. **(C)** Western blotting of recombinant human IL-4 (rhIL-4), control, and staurosporine-treated cell lysates demonstrate that the protein(s) upregulated by staurosporine treatment is of high molecular weight, with a major band around 100 kD (black arrow). Western blot data are representative of 4 stimulations and experiments from 4 donors.

Previous studies have demonstrated that IL-4 expression is associated with apoptosis, and these studies share the usage of 8D4-8 (Ledru et al., 2003; Veenstra et al., 2008). We sought to investigate this apoptosis-associated IL-4 and treated PBLs with increasing concentrations of the apoptosis-inducer staurosporine (Belmokhtar et al., 2001). We then evaluated IL-4 (8D4-8) expression by flow cytometry. These cells were not given PTIs as a previous report indicated this IL-4 variant is not secreted (Ledru et al., 2003). We observed robust increases in 8D4-8+ cells with increasing levels of staurosporine concentrations, thus replicating previous work (Figure 4A,B). Irrespective of treatments, 8D4-8+ cells were largely Live/Dead+ (Figure 4A). Using this method as a way to enrich for IL-4 expression, we then prepared lysates from staurosporine-treated and control lymphocytes and compared them by western blot. We observed substantial increases in high molecular weight bands (∼100 and ∼250 kD) with staurosporine treatment compared to control (Figure 4C). These data matched what we observed with staurosporine-treated cells using flow cytometry and 8D4-8. Thus, we concluded that the additional protein detected by 8D4-8 was ∼100 kD or greater, well out of range for any known IL-4 isoform.

**FIGURE 5:**
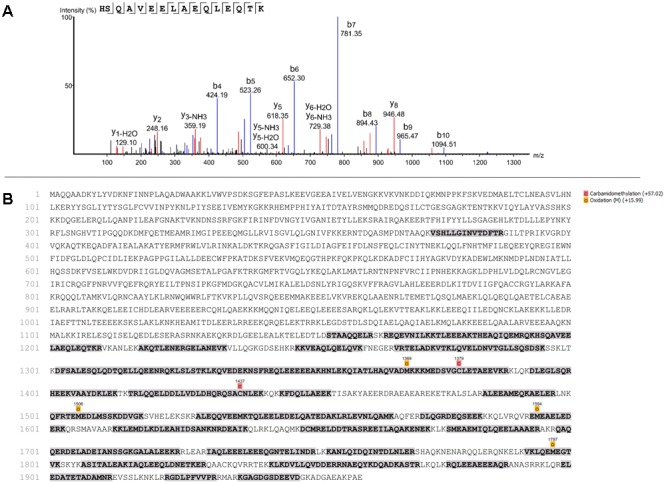
Mass spectrometry showing identification of peptide and sequence coverage of myosin-9. Results from one mass spectrometry analysis of the ∼100 kD band from an 8D4-8 immunoprecipitation of staurosporine-treated PBL. In total, 2 control and 2 staurosporine-treated samples were analyzed from 2 separate experiments. **(A)** Identification of peptide representing sequence from 1194 to 1209 of myosin-9. **(B)** Sequence coverage (31%) of myosin-9.

**FIGURE 6:**
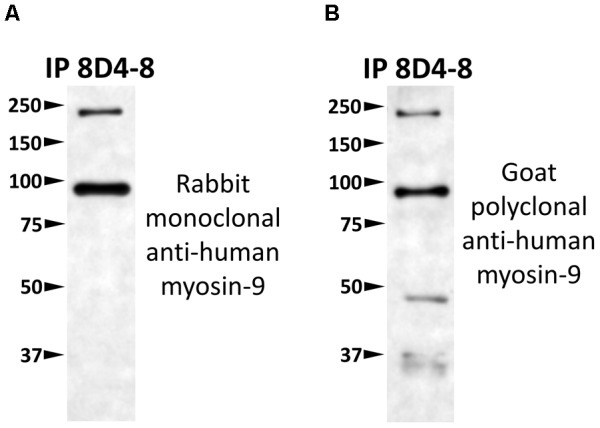
8D4-8 immunoprecipitates myosin-9. We western blotted equal volumes of 8D4-8 immunoprecipate and targeted with either **(A)** rabbit monoclonal anti-myosin-9, or **(B)** goat polyclonal anti-myosin-9. Both myosin-9 antibodies detected bands at ∼225 and ∼95 kD, in the size range of full length myosin-9 as well as the apoptosis-associated tail piece. Data are representative of 2 separate immunoprecipitations of control and staurosporine-treated lysates which were used for mass spectrometry analysis.

To identify which protein 8D4-8 was binding, we performed immunoprecipitations(IPs) on control or staurosporine-treated lymphocytes. We then excised bands at ∼100 kD and identified the proteins via mass spectrometry. Our data indicated both control (data not shown) and staurosporine IPs (Figure 5A,B) were enriched for a tail section of myosin-9 in the 100 kD region. We then blotted IPs from control and staurosporine treated cells with commercial antibodies specific to tail regions of myosin-9. Anti-myosin-9 goat polyclonal antibody EB09020 was generated against the sequence DQINTDLNLERSH (aa 1760–1762), while anti-myosin 9 rabbit monoclonal antibody EPR8965 was generated against a proprietary sequence between aa 1900–2000 of full length myosin-9. Using these antibodies, we observed strong bands at ∼225 and ∼95 kD with both antibodies from both control (data not shown) and staurosporine-treated lysates (Figure 6). These results demonstrated that 8D4-8 cross-reacts with myosin-9. It has been previously published that the myosin-9 tail breaks away during apoptosis (Suarez-Herta et al., 2000; Kato et al., 2005). Judging from our ability to detect the ∼95 kD fragment in whole cell lysates and following IP, it’s likely that 8D4-8 binds somewhere along the tail region.

**FIGURE 7:**
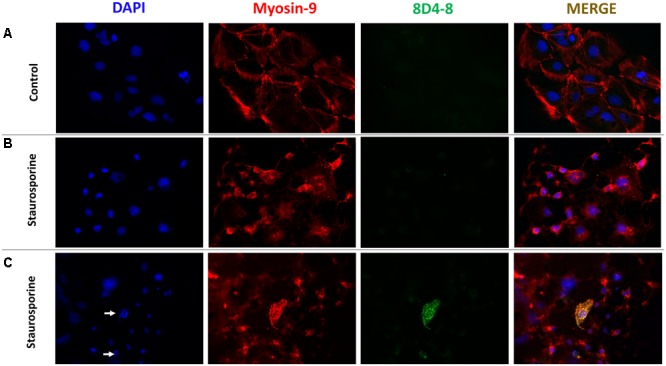
8D4-8 and myosin-9 colocalize among apoptotic cells. **(A)** We observed no detectable 8D4-8 expression among healthy control Caco-2 cells. **(B)** The majority of staurosporine-treated Caco-2 cells show retracted processes and more condensed cytoplasm, but do not bind 8D4-8. **(C)** Scattered among the staurosporine-treated cells were 8D4-8+ cells. These cells were limited to those in apparent apoptosis. Note the condensed chromatin (white arrows) associated with the 8D4-8+ cells. For **(A–C)**, blue is DAPI, red is myosin-9, and green is 8D4-8. Images were acquired at 200× magnification. Results are representative of 3 experiments.

To follow up on these findings, we used immunofluoresence to query coexpression of 8D4-8 and myosin-9. Using Caco-2 cells, a colorectal adenocarcinoma cell line, we observed broad specific myosin-9 expression with no 8D4-8 binding in untreated cells (Figure 7A). Following overnight treatment with staurosporine, the majority of adherent cells had exhibited retracted processes and condensed cytoplasm yet no binding of 8D4-8 (Figure 7B). Scattered throughout, in various stages of disassociation, we found Caco-2 cells advanced in apoptosis which exhibited striking 8D4-8 expression that colocalized with myosin-9 (Figure 7C). This coexpression was limited to cells with condensed or fragmented nuclei (Figure 7C white arrows).

**FIGURE 8:**
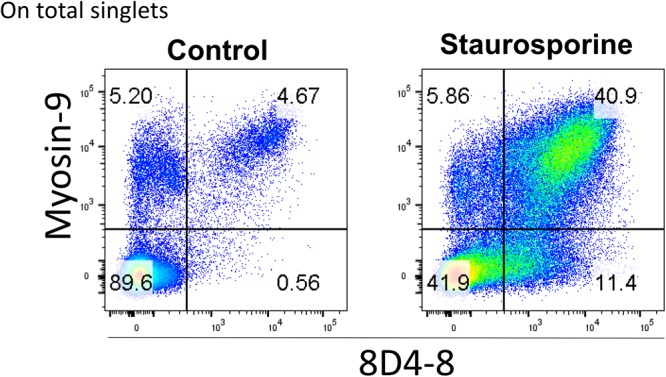
Surface detection of 8D4-8 and myosin-9 colabels a population of increased size following staurosporine treatment. PBL were pregated to remove doublets (not shown). Cellular permeabilization is not required to detect 8D4-8 and myosin-9 among control or staurosporine-treated PBL. Results are representative of 3 experiments from 3 donors.

Finally, it’s been reported that myosin-9 is expressed externally on the surface of apoptotic cells and can be detected with anti-myosin-9 antibodies without cellular permeabilization (Chu et al., 2010). We reasoned that since 8D4-8 detects myosin-9 associated with apoptosis, we should be able to detect 8D4-8+, myosin-9+ (double-positive) events on non-permeabilized lymphocytes. We then performed surface flow cytometry using 8D4-8 and anti-myosin-9 antibodies to test this. As previously reported, myosin-9 was found to be accessible without permeabilization, and this was also the case for 8D4-8 (Figure 8). In both staurosporine-treated and control PBL, these double-positive events demonstrated somewhat linear expression levels. Furthermore, we observed increased proportions of double-positive events among staurosporine-treated PBL compared to control. These observations provide further evidence of 8D4-8 binding myosin-9.

## Discussion

During the process of routine flow cytometry panel validation, we observed unique behavior associated with a widely available mouse monoclonal antibody targeting human IL-4, clone 8D4-8. This is not the first report of such unique results using this clone. From previously published literature, the application of clone 8D4-8 in flow cytometry experiments can be divided into two groups: those cognizant of 8D4-8 detecting an apoptosis-associated IL-4 and those unaware that 8D4-8 responds uniquely among apoptotic cells. Among the former group, the earliest reports demonstrated increased IL-4 expression following various apoptosis inducers among human PBMC and several cell lines (Stein et al., 2000a,b). The concept was further developed with the report that the 8D4-8 target was not secreted and was linked to a unique IL-4 mRNA missing 13 bp, dubbed IL-4δ_13_ (Ledru et al., 2003). Increased 8D4-8 expression among apoptotic cells was also observed among all major classes of circulating leukocytes (Veenstra et al., 2008), as well as among γδT cells (Bhat et al., 2016). Additionally, elevated 8D4-8 expression was associated with resting T cells of early childhood (Hebel et al., 2014). Our results indicate that these studies were likely observing myosin-9 rather than IL-4. Importantly, we were unable to detect any bands in the range of purported IL-4 size with 8D4-8 following staurosporine treatment. Rather, we observed enriched abundance of a ∼100 kD target which was found to be a chain of myosin-9 that was associated with apoptosis. Colocalization immunofluorescence experiments indicated that 8D4-8 only significantly reacted with myosin-9 among apoptotic cells, while dual surface labeling of 8D4-8 and myosin-9 on staurosporine-treated cells demonstrated a high level of double positive events. In total, we have found no evidence to support the hypothesis that apoptotic cells produce substantial amounts of IL-4 of any isoform.

The application of 8D4-8 in studies where users did not know it bound a unique apoptosis-associated target presents a sizable issue. In evaluating the literature for IL-4 expression measured by flow cytometry in the time since the publication of 8D4-8, we found multiple publications reported using this clone (Prussin and Metcalfe, 1995; Fukui et al., 1997; Kallas et al., 1998; Kon et al., 1998; Schulze-Koops et al., 1998; Furlan et al., 2000; Kipp et al., 2000; Johansson-Lindbom and Borrebaeck, 2002; Rutella et al., 2002; Ryan et al., 2002; Sugimoto et al., 2002; Walker et al., 2002; Hashizume et al., 2005; Kilani et al., 2005; Nakamura et al., 2005; Acosta-Rodriguez et al., 2007; Ito et al., 2008; Delgado et al., 2009; Godefroy et al., 2011; Saito et al., 2011; Truchetet et al., 2011; Caccamo et al., 2012; Miguel et al., 2012; Baeck et al., 2013; LaVoy et al., 2013; Myklebust et al., 2013; Qiu et al., 2013; Jergovi et al., 2014; Mattoo et al., 2014; Periolo et al., 2014; MacDonald et al., 2015; Wang S. et al., 2015; Wang Z. et al., 2015; Coraglia et al., 2016; De Biasi et al., 2016; Li et al., 2016; Vacaflores et al., 2016; Wang et al., 2016; Hou et al., 2017; Powell et al., 2017; Rouxel et al., 2017; Fox et al., 2018; Kim et al., 2018; Kwok et al., 2018; Maselli et al., 2018; Rotolo et al., 2018; Sureshchandra et al., 2018; Mann et al., 2019; Schreurs et al., 2019). This list should not be considered exhaustive as some publications may have been missed and many reports evaluating IL-4 did not mention which clone was used. Regarding the known literature for 8D4-8 detecting canonical IL-4 expression, it must be reevaluated carefully. While our data indicate that 8D4-8 can truly bind IL-4, this can only be completely confirmed in flow cytometry by using an additional unique clone, an approach which is rarely taken. Furthermore, our data indicate that even the use of a dead cell discriminator will not completely eliminate myosin-9 binding from an 8D4-8 experiment. Finally, the ability for 8D4-8 to bind apoptotic, resting targets both intracellularly and on the cellular surface presents grave technical obstacles as “IL-4+” cells can be measured in the absence of proper cellular activation or functional cellular permeabilization. Thus, in total, we conclude that 8D4-8 should not be used in flow cytometric applications interrogating IL-4 expression. In our hands, clones 4D9 and REA895 were the best choices for IL-4 expression experiments, although clones MP4-25D2 and 3010.211 were acceptable. While untested here, clone 8D4-8 may still be suitable for IL-4 ELISA and ELISpot, and can be used with caution in western blotting. Alternatively, it could be repurposed to evaluate myosin-9 breakdown in apoptotic cells.

Being beyond the scope of our goal of panel and antibody validation, the mechanism behind 8D4-8 binding myosin-9 remains unresolved. We observed functional binding of 8D4-8 in several scenarios: (1) reducing and non-reducing (data not shown) denaturing conditions, (2) following formalin fixation, (3) and on unfixed cells. In total, these results suggest an exposed linear epitope becomes available as cells progress through the stages of apoptosis. Previous work on myosin-9 degradation associated with apoptosis demonstrated two cleavage sites at Asp-1153 and Asp-1948 resulting in a 95 kD fragment (Kato et al., 2005). In sequence aligning of IL-4 and myosin-9 aa 1153–1948, we observed a handful of similarities which could be binding regions for 8D4-8 (data not shown). However, epitope mapping within this region should be performed to properly define the binding sequence.

In conclusion, the process of flow cytometry panel validation requires multiple steps in order to generate successful assays (Mahnke and Roederer, 2007). Being (generally) an antibody-based approach, the assessment of antibody specificity must be included in the validation process as well. While this increases panel validation time and cost, the outcome of comparing the performance of a handful of clones is confidence in the reproducibility and integrity of the assay – certainly worthy recompense. Of course, this is not a novel suggestion. The problems of antibody specificity, validation, and the associated “reproducibility crisis” have been on display for some time (Saper, 2009; Voskuil, 2014; Baker, 2015; Weller, 2016). The methods to correct these problems are not infeasible, they just require their incorporation into all optimization workflows.

## Author Contributions

RH designed and performed the experiments, analyzed the data, and wrote the manuscript. KB performed the experiments and analyzed the data. VK performed the mass spectrometry analysis. NS designed the experiments.

## Conflict of Interest Statement

The authors declare that the research was conducted in the absence of any commercial or financial relationships that could be construed as a potential conflict of interest.
